# An Optimized and Cost-Effective RNA Extraction Method for Secondary Metabolite-Enriched Tissues of Norway Spruce (*Picea abies*)

**DOI:** 10.3390/plants13030389

**Published:** 2024-01-28

**Authors:** Vivek Vikram Singh, Aisha Naseer, Gothandapani Sellamuthu, Rastislav Jakuš

**Affiliations:** 1 Faculty of Forestry and Wood Sciences, Czech University of Life Sciences Prague, Kamýcká 129, Praha-Suchdol, 165 00 Prague, Czech Republic; naseer@fld.czu.cz (A.N.); sellamuthu@fld.czu.cz (G.S.); jakus@fld.czu.cz (R.J.); 2 Institute of Forest Ecology, Slovak Academy of Sciences, Štúrova 2, 960 53 Zvolen, Slovakia

**Keywords:** RNA extraction kits, TRIzol, CTAB, nanodrop, RNA integrity, phenolic content, RNA-Seq

## Abstract

Since the development of next-generation sequencing techniques and with the growing interest in transcriptomic studies, there is a demand for high-throughput RNA extraction techniques. General RNA extraction protocols are unreliable when it comes to the quality and quantity of isolated RNA obtained from different tissue types of different plant species. Despite Norway spruce (*Picea abies*) being one of the most significant and commercially valuable tree species in European forests, only limited genetic research is available. In this study, we developed a cetyltrimethylammonium bromide (CTAB) protocol by modifying the original method. We compared this CTAB protocol with other widely used methods for extracting RNA from different tissues (needle, phloem, and root) of Norway spruce, known for its richness in polyphenols, polysaccharides, and secondary metabolites. The modified CTAB method proves to be superior to the kit-based and TRIzol-based methods for extracting RNA from the metabolite-rich tissues of Norway spruce, resulting in high RNA quality and integrity values (RIN~7–9). The modified CTAB RNA extraction method is rapid, cost-effective, and relatively simple in yielding the desired RNA quality from Norway spruce tissues. It is optimal for RNA sequencing and other downstream molecular applications.

## 1. Introduction

Norway spruce (*Picea abies* [L.] Karst) is one of the most important species in European boreal forests and serves as the economic backbone of the timber industry in many European countries. Ongoing climate change has had a negative impact on the Norway spruce population in the forest [1]. The rise in spruce mortality over the past few decades can be attributed to a combination of biotic factors, such as bark beetle (*Ips typographus*) outbreaks and fungal pathogens, and abiotic factors, including drought and windthrows, or a combination of both [2,3,4]. In particular, bark beetle outbreaks are often triggered by environmental factors, such as droughts and increased temperatures, resulting in compromised tree defense. The current traditional management practices have not been successful in eliminating the calamity. Therefore, understanding the molecular mechanisms of spruce survival and resilience towards abiotic and biotic factors is essential to further develop and design novel management practices.

Advances in next-generation sequencing technologies and bioinformatics have transformed our ability to explore the biology of plant growth and its reaction to environmental stimuli and stresses [5,6,7]. Understanding the fundamental processes of gene expression, signal transduction, gene regulation, and transcriptome analysis involves employing a diverse set of methodologies, including RNA sequencing (RNA-Seq), reverse transcription polymerase chain reaction (RT-PCR), and complementary DNA (cDNA) library preparation [8,9]. However, obtaining good-quality RNA is of prime necessity for carrying out RNA-Seq analyses, which are used for gene expression studies in different tissues and plants under stress, detecting mutations, alternative splicing, and post-transcriptional modifications.

Plants exhibit a wide range of biochemical compositions of secondary metabolites, posing a challenge in obtaining RNA of the desired quality for subsequent downstream molecular applications [10,11,12]. The complexity of plant biology extends beyond the plant’s biochemical profile, encompassing factors such as growth stage, physiological status, tissue type, and environmental conditions. In addition, the extraction of high-quality RNA is further complicated by variations in plant species (due to their anatomical and biochemical profiles), even within the same genus, necessitating protocol optimization. In coniferous species, the low number of living cells in tissues and their highly lignified nature make cell disruption difficult [13]. Furthermore, high concentrations of polysaccharides, phenolics, and RNase-associated contaminations make isolating good-quality RNA difficult [8,14,15]. The phenolic compounds in tissues undergo oxidation to form quinones, which can irreversibly bind to nucleic acids and co-precipitate with RNA, ultimately resulting in RNA degradation and a low yield [5,8].

Different plant RNA extraction protocols employ guanidinium-thiocyanate-based, cetyltrimethylammonium bromide (CTAB)-based, or sodium dodecyl sulfate (SDS)-based approaches. However, it is crucial to optimize these methods for individual species and even for the characteristics of the target tissue [15,16]. Commercially available preparations use guanidium isothiocyanate and phenols to provide a high yield, but the quality remains low and is useful only for certain model plant systems [17]. Successful RNA extraction using CTAB, TRIzol, and TRIzol plus RNA Purification kits have been documented [9,16,18,19,20,21,22,23,24], but they are often unsuitable for recalcitrant and diverse plant tissues. In addition, several methods and their variations have been described for extracting RNA from waxier and high secondary metabolite-containing plant tissues [14,22,25]; however, they are time-consuming, cumbersome, and only work with specific tissue types.

In this study, we compared widely used approaches and described a validated method for extracting RNA from different Norway spruce tissue types (needle, phloem, and root) for RNA-Seq. The TRIzol-based methods (standard TRIzol and TRIzol-column) yielded no significant amount of RNA, whereas the Qiagen RNesay^®^ kit and Sigma-Aldrich kit were able to extract a moderate quantity of RNA from specific tissue types only. The described CTAB method is a preliminary modification of the standard CTAB protocol reported by Chang et al. [14] and has proven to be highly efficient in obtaining pure, intact, and high amounts of RNA from Norway spruce tissues. The protocol is short, simple, and economically feasible. The quality of the isolated RNA is consistently high, as evidenced by spectrophotometric readings and its separation in agarose electrophoresis gel.

## 2. Results and Discussion

**Figure 1:**
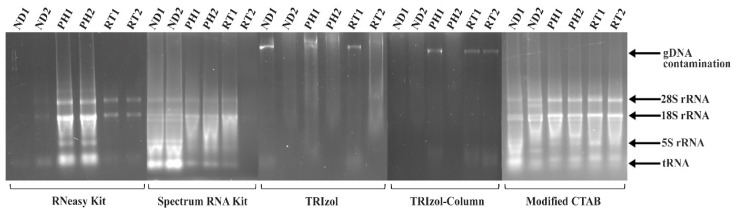
Agarose gel electrophoresis (AGE) of RNA isolated from needle (ND), phloem (PH), and root (RT) tissues using different RNA isolation methods, i.e., RNeasy^®^ Plant Mini Kit, Spectrum Plant Total RNA Kit, standard TRIzol, TRIzol-column hybrid, and the modified CTAB protocol (numbers after tissue name, viz. 1–2 indicate biological controls). gDNA shows genomic contamination of the sample isolate. The RNA yield and absorbance ratios are mentioned in Table 1. To facilitate comparison, an equal volume of RNA (4 µL) was loaded due to the unavailability of sufficient RNA concentration in certain samples to be loaded in equal amounts (50 or 100 ng).

Successful RNA isolation is a crucial step in fundamental and applied research in molecular biology. Isolating high-quality RNA is essential for accurate downstream analyses, including gene expression studies, microarray analysis, RT-PCR, and RNA-Seq [15,26,27]. Obtaining a high-quality and quantity of RNA can be a significant challenge, and it often requires careful consideration of several factors [28]. The secondary metabolites, polysaccharides, and polyphenolic compounds present in conifers [29] can interfere with the RNA isolation process, leading to degraded RNA and resulting in low RNA yield and poor quality [5,8]. This study tested the efficiency of five widely used RNA extraction methods for various tissue types of Norway spruce. The modified CTAB method, based on Chang et al. [14], was compared with commercial preparations (RNeasy^®^ Plant Mini Kit, Spectrum Plant Total RNA Kit), the standard TRIzol method, and the TRIzol-column hybrid method [22]. The modified CTAB method exhibited the best results in both RNA quality and quantity (Figure 1 and Table 1).

The RNeasy^®^ Plant Mini Kit yielded inadequate RNA concentrations in phloem samples, and nanodrop readings indicated negligible yields in needle and root samples. Although agarose gel electrophoresis (AGE) revealed visible RNA bands in phloem samples and weak bands in root samples, the quantity of RNA was found to be insufficient for downstream applications (<10 ng/µL) (Table 1). Previous studies have demonstrated the low quality and quantity of RNA isolated from *Corylus avellana* L. (hazelnut) and *Psidium guajava* L. (guava) using commercial kits [30,31]. Conversely, the Spectrum Plant Total RNA Kit produced acceptable nanodrop readings for needle tissue samples but yielded low concentrations in phloem and root samples. The AGE showed visible but weak rRNA bands at the 28S and 18S regions, with significant smearing at the 5S rRNA region, suggesting RNA degradation. This observation indicates that this method could be suitable for needle tissue but not phloem and root tissue due to poor yield (Table 1). Similar results were reported with much lower RNA yields from white spruce (*Picea glauca*) and white pine (*Pinus strobus* L.) compared to other woody plants [32].

The TRIzol reagent is a strong phenol-containing lysis solution that helps to disrupt cells while maintaining RNA integrity. However, in our study, the TRIzol-based extraction methods, including the standard TRIzol and the TRIzol-column hybrid approach, proved ineffective for isolating RNA from any tissue type of Norway spruce [15,33]. AGE showed the absence of visible bands in the RNA region, marked by substantial smearing across all lanes. Interestingly, nanodrop readings indicate the presence of nucleic acids for the TRIzol method; however, the gel displayed no visible bands in the RNA region. Notably, the TRIzol isolation procedure often resulted in browning of the extract, a phenomenon frequently reported by others and is usually accompanied by RNA damage caused by high concentrations of polyphenolics present in conifer tissues [13,14,34]. This discoloration is typically attributed to the co-precipitation of polyphenols, leading to a low RNA yield. Analysis of the 260/280 ratio, 260/230 ratio, and nanodrop nucleic acid concentrations suggests significant contamination in the RNA samples isolated using the kit and TRIzol methods (Table 1). Other studies have isolated low-quality RNA from *P. guajava* and *Vitis vinifera* using the TRIzol method [31,32].

**Figure 2:**
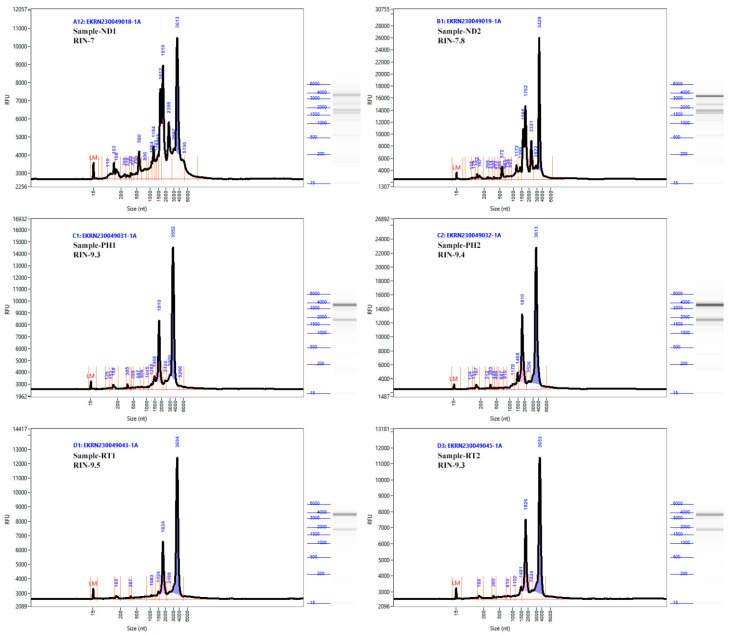
Agilent 5400 Fragment Analyzer System assessed RNA integrity test results of RNA samples extracted using the modified CTAB method (adapted from the NovoGene pre-sequencing report).

The CTAB method consistently yielded superior RNA quality, as evidenced by the nanodrop readings (Table 1, Figure S1), and showed clearly visible 28S and 18S rRNA bands with a slightly visible smear at the 5S rRNA region in AGE (Figure 1). The 260/280 and 260/230 ratios are close to 2 and 1.9, respectively, suggesting no considerable genomic DNA and protein contamination. One of the needle replicates (ND1) exhibited a relatively weak band in the gel with visible RNA degradation. However, this did not compromise the amount and quality of RNA, as reflected in the pre-sequencing quality-check report (refer to Table 2 and Figure 2). The observed degradation is possibly due to handling errors.

These results show that the CTAB method is most suitable for extracting RNA from all tissue types of Norway spruce and can be used as a standard procedure for downstream applications. In contrast, other protocols were only successful with specific tissue types and failed to isolate high-quality RNA across all tissue types. Our findings are consistent with other studies that demonstrated high quality and high yields of RNA obtained from different tissues of *P. guajava* [31], *Kandelia candel*, *Rhizophora mucronata*, *Oryza sativa*, *Hordeum vulgare*, *Brassica napus*, *Juglans regia*, *Malus domestica*, *Solanum tuberosum* L., and many other recalcitrant species [35,36].

Pre-sequencing RNA sample quality and quantity tests were performed using nanodrop and the Agilent 5400 Fragment Analyzer System (refer to Table 1 and Figure 2). Despite the high intensity of the 5S region and some consistent signal patterns at the 18S region, which are not ideal according to the RIN standards, the sequencing outcome suggests otherwise. The RNA extracted from various tissues of Norway spruce using the modified CTAB method produced good results, as all the samples exceeded the defined criteria for good-quality data in next-generation sequencing, with a minimum quality score of Q20 ≥ 98 and Q30 ≥ 94 (Table 2).

Several protocols have been developed for RNA isolation from conifers, with a few specifically optimized for needles [14,37,38], phloem [34], and roots [14]. With the aim of minimizing both the time and cost of extraction without reducing the quality and yield of RNA, we modified the CTAB protocol by introducing several changes to the method originally outlined by Chang et al. [14]. The described protocol efficiently isolates RNA from various tissues, yielding higher concentrations (see Table 1). Notably, the key divergence from the original protocol is the use of a 1-h precipitation step instead of overnight precipitation. This modification significantly expedites the procedure without compromising the final RNA quality and yield. Additionally, the described method streamlines the ethanol washing of the RNA pellet, requiring less than 2 min. In contrast, the original protocol suggests keeping the supernatant with ethanol for between 30 min and 2 h at −20 °C. These adjustments collectively enhance the efficiency of the RNA isolation process while maintaining reliable results. The major problem in dealing with secondary metabolite-enriched tissues is the oxidation of phenolic compounds, which form quinone/protein complexes and often co-precipitate with RNA [13,15]. This results in poor quality and a low yield. To overcome this problem, the use of PVP and BME as reducing reagents has been suggested [14]. We used CTAB as a detergent and performed extraction with chloroform:Isoamyl (24:1) alcohol instead of phenol to remove proteins. Repeating this step allowed us to remove the excess CTAB and polysaccharides during the chloroform:Isoamyl extraction phase. The use of a high concentration of NaCl in the extraction buffer helped to eliminate the polysaccharides. Further, LiCl was used for precipitation to obtain a colorless RNA pellet. LiCl helps in the efficient precipitation of RNA by excluding unincorporated or smaller nucleotide particles, such as DNA and proteins, from the solution [39]. The quality of the obtained RNA using this method was excellent, and poly A(+)-RNA damage was minimized by avoiding phenol-mediated extraction [14].

## 3. Material and Methods

### 3.1. Plant Material and Sampling

Norway spruce needle, phloem, and root samples were collected and snap-frozen in liquid nitrogen on-site to prevent RNA degradation. The age of the selected tissues influences RNA yield and purity; therefore, young needles and fine roots were selected from 4-year-old Norway spruce plants. In all the protocols, the tissue samples were ground to a fine powder in liquid nitrogen using a pre-chilled mortar and pestle. The ground tissue powder was used immediately or was stored at −80 °C for later use. All five applied protocols used two biological replicates per tissue.

### 3.2. Total RNA Extraction

All the reagents were prepared in RNase-free water, and the glasses, mortars, and pestles were autoclaved and then heat dried overnight at 60 °C. All the samples were derived from the same source material and in the same quantity. Two different commercial kit-based methods, i.e., the RNeasy^®^ Plant Mini Kit (Qiagen, Hilden, Germany) and the Spectrum Plant Total RNA Kit (Sigma-Aldrich, St. Louis, MO, USA), and three manual methods, i.e., the standard TRIzol^®^ (Ambion, Foster City, CA, USA), the TRIzol-column hybrid [22], and the modified CTAB method, were compared to determine the most suitable method for RNA extraction from Norway spruce.

#### 3.2.1. RNeasy^®^ Plant Mini Kit Protocol

Total RNA extraction was performed using the RNeasy^®^ Plant Mini Kit (Qiagen, Germany), following the manufacturer’s instructions. Briefly, 100 mg of homogenized tissue was added to 450 µL of RLT buffer that was pre-prepared by mixing β-mercaptoethanol (BME), as per the instructions. The mixture was vortexed vigorously, and lysate was transferred to a QIAshredder spin column, placed in a 2 mL collection tube, and centrifuged at maximum speed for 2 min. Then, 0.5 volume of absolute ethanol was added to clear lysate and was mixed gently. The sample was transferred to a RNeasy Mini spin column and was centrifuged for 15 s at 10,000 rpm, and the flow-through was discarded. RW1 buffer (700 µL) was added to the RNeasy spin column and was centrifuged at 10,000 rpm for 15 s. Then, 500 µL of buffer RPE was added to the column and centrifuged, followed by flow-through discard. The buffer RPE step was repeated once. The column was dry centrifuged for 1 min. Finally, the RNeasy column was placed in a collection tube, and RNase-free water (~50 µL) was added to the column membrane, followed by 1 min of centrifugation to elute the RNA.

#### 3.2.2. Spectrum Plant Total RNA Kit Protocol

Total RNA extraction using the Spectrum Plant Total RNA Kit (Sigma-Aldrich, USA) was performed as per the manufacturer’s instructions. First, 500 µL of lysis solution/BME mixture was added to 100 mg of finely grounded tissue and was vortexed vigorously for 30 s. The sample was then incubated at 56 °C for 3–5 min. Then, the sample was centrifuged for 3 min at maximum speed. The supernatant was filtered into a filtration column and was seated onto a collection tube. The lysate was centrifuged for 1 min at maximum speed to remove the residual debris. The clarified flow-through was collected, and 250 µL of binding solution was added and mixed thoroughly by pipetting. Then, the mixture was added to the binding column and was centrifuged for 1 min at 12,000 rpm to bind the RNA. The flow-through was decanted. A total of 500 µL of wash solution 1 was added to the column and was centrifuged for 1 min, and the flow-through liquid was decanted. This step was repeated twice with wash solution 2 and 30 s of centrifugation. After discarding the flow-through, the column was dry centrifuged for 1 min at 12,000 rpm to remove the residual flow-through liquid. Finally, the column was transferred to a new 2 mL collection tube and was eluted by adding 50 µL of elution solution to the center of the binding matrix inside the column.

#### 3.2.3. TRIzol Protocol

Homogenized tissue samples (100 mg) were transferred into 1 mL of TRIzol in a 2 mL microcentrifuge tube. A total of 10 µL of fresh BME solution was added, mixed well by vigorous shaking/vortexing, and incubated for 5 min at room temperature. The tube was centrifuged at 12,000 rpm for 10 min at 4 °C. The aqueous phase was transferred into a 1.5 mL microcentrifuge tube and 200 µL of chloroform was added. The sample was mixed well, incubated for 5 min at room temperature, and centrifuged for 10 min at 12,000 rpm for phase separation. The aqueous phase was transferred into a 1.5 mL microcentrifuge tube and 0.7 volume of chilled isopropanol was added. The solution was mixed well by inversion and was incubated for 1 h at −20 °C. The supernatant was decanted after centrifugation at 12,000 rpm for 10 min at 4 °C. The pellet was washed with 70% ethanol. The supernatant was gently poured off or removed using a pipette. This step was repeated. The pellet was air dried and eluted in 50 µL of RNase-free water.

#### 3.2.4. TRIzol-Column Hybrid Protocol

The TRIzol-column hybrid method was used to extract RNA from needles, phloem, and root tissues following Untergasser’s protocol [22]. First, 500 µL of TRIzol was added to homogenized tissue and was incubated for 5 min at room temperature. Then, 100 µL of chloroform was added and mixed well, and the mixture was incubated for 2 min at room temperature. The mixture was then centrifuged at 12,000 rpm for 15 min at 4 °C. The aqueous upper phase was transferred to a new tube, and an equal volume of 70% ethanol was added and mixed well. The phase liquid was transferred to the RNeasy Mini spin column and was centrifuged at 12,000 rpm for 15 s, and the flow-through was discarded. Then, 350 µL of buffer RW1 was added to the column and centrifuged, and the flow-through was discarded. A total of 500 µL of buffer RPE was added to the column and centrifuged, and the flow-through was discarded. The buffer RPE step was repeated. The column was placed in a fresh collection tube and was dry centrifuged for 2 min at maximum speed. Finally, the column was placed in a new microcentrifuge tube and eluted with the appropriate amount (~50 µL) of RNase-free water.

#### 3.2.5. Modified CTAB Method

**Figure 3:**
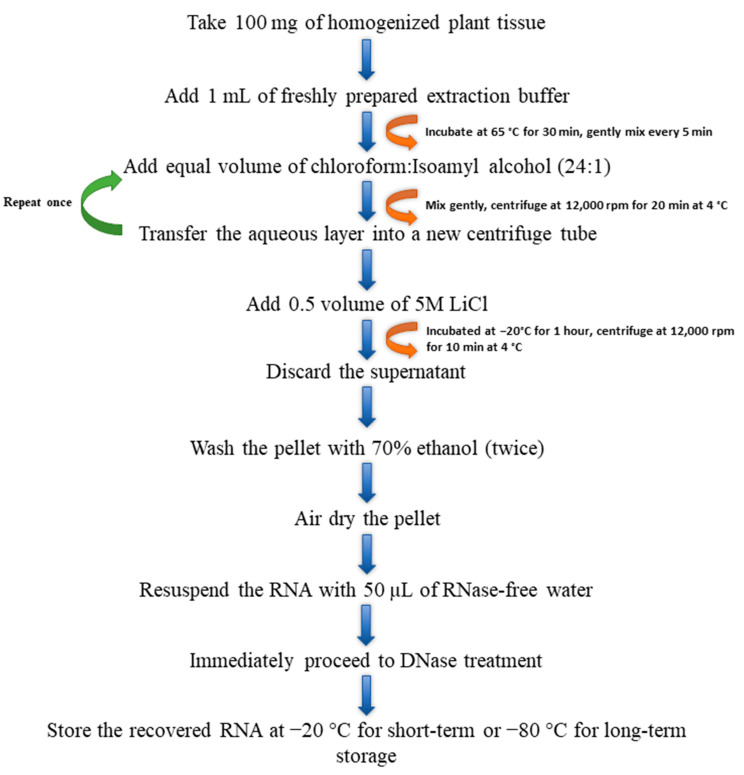
Flow chart describing the steps involved in total RNA extraction using the modified cetyltrimethylammonium bromide (CTAB) method.

The CTAB protocol was modified from Chang et al. [14]. The extraction buffer was composed of 2.0% (*w*/*v*) CTAB (Sigma-Aldrich, St. Louis, MO, USA), 2.0% (*w*/*v*) polyvinylpyrrolidone (PVP 40; Sigma-Aldrich, St. Louis, MO, USA), 2M sodium chloride (NaCl, Sigma-Aldrich, St. Louis, MO, USA), 100 mM Tris-HCl (pH 8.0; Sigma-Aldrich, St. Louis, MO, USA), 25 mM ethylenediaminetetraacetic acid (EDTA; Sigma-Aldrich, St. Louis, MO, USA), and 2.0% (*w*/*v*) β-mercaptoethanol (BME, Sigma-Aldrich, St. Louis, MO, USA) in RNase-free water. The components in the extraction buffer, excluding BME and Proteinase K (Ambion, Foster City, CA, USA), were mixed and pre-warmed at 65 °C for 10 min. Then, 2% BME and 80 μg/mL of Proteinase K were added before RNA extraction. The homogenized tissue samples (100 mg) were transferred into 1 mL of extraction buffer and were incubated at 65 °C for 30 min, and the samples were mixed at 5-min intervals. An equal volume of chloroform:Isoamyl alcohol (24:1) was added into the mixture, mixed, and centrifuged at 12,000 rpm for 10 min at 4 °C. The chloroform:Isoamyl alcohol step was repeated once. The top aqueous layer from the tube was transferred into a new 2 mL centrifuge tube. Then, 0.5 volume of 5M lithium chloride (LiCl) was added, mixed, and incubated at −20 °C for 1 h. The mixture was centrifuged at 12,000 rpm for 10 min at 4 °C. The supernatant from the tube was discarded, and the pellet was washed twice with 70% ethanol. The pellet was air dried and eluted with an appropriate amount of RNase-free water (~50 µL) (Figure 3).

### 3.3. DNase Treatment and RNA Analysis

All the described protocols were subjected to DNase treatment after RNA extraction and subsequent DNA-digestion to remove residual DNA contamination. The TURBO DNA-free™ Kit (Invitrogen, Carlsbad, CA, USA) was used to treat the isolated RNA, as per the protocol. Briefly, TURBO DNase buffer (1×) and 2 units of TURBO DNase enzyme were added to the sample and incubated at 37 °C for 30 min. The DNase-treated RNA was then used to check the integrity and purity. RNA purity was verified using the NanoDrop2000 spectrophotometer (Thermo Scientific, Waltham, MA, USA). The OD260/OD230 and OD260/OD280 ratios were measured, with a higher or closer value to 2 indicating purer RNA in the sample. RNA integrity was verified by running the samples on 1.5% agarose gel electrophoresis (AGE). Good-quality RNA samples were represented by intact 28S and 18S rRNA bands without smearing.

### 3.4. RNA Sequencing

The RNA extracted from Norway spruce tissues using the modified CTAB method was dissolved in RNase-free water and shipped for sequencing at NovoGene (NovoGene, Beijing, China). The Agilent 5400 platform (Agilent, Santa Rosa, CA, USA) was used to check sample quality and integrity before sequencing, and the Illumina sequencing platform was used for pair-end sequencing. Based on the ratio of the large (28S) to small (18S) ribosomal RNA subunits (28S/18S), the RNA integrity number (RIN) was calculated and used to determine the quality of the RNA sample.

## 4. Conclusions

This study systematically assessed five distinct RNA extraction methods, comprising two commercially available kit-based approaches (Spectrum and Qiagen kits) and three manual methods (TRIzol, TRIzol-column hybrid, and modified CTAB), based on the best RNA quality and quantity isolated from different Norway spruce tissue types. Although the kits are commercially available and relatively easy to use, they are not suitable for all tissue types and are not cost-effective. Although the TRIzol reagent solution appeared to work with certain tissues based on nanodrop readings, the RNA purity and integrity were highly compromised. Overall, the modified CTAB method consistently delivered superior results, demonstrating high-quality RNA and robust yields. It emerged as the most suitable and cost-effective choice for RNA extraction from Norway spruce tissues. The versatility of the modified CTAB method is evident, as it proved effective across various tissues. This method can be used without further modification to obtain a good yield for future sequencing-based studies of Norway spruce and other woody plants.

## Figures and Tables

**Table 1:** Norway spruce tissue RNA extract quality and quantity assay using the RNeasy^®^ Plant Mini Kit, Spectrum Plant Total RNA Kit, TRIzol, TRIzol-column hybrid, and modified CTAB methods using a NanoDrop2000 spectrophotometer (Thermo Scientific, USA).

		Absorbance Ratio		
Method	Replicate	260/280	260/230	RNA Concentration (ng/µL)
RNeasy^®^ Plant Mini Kit	ND1	1.69	0.02	4.2
	ND2	1.68	0.09	5.3
	PH1	1.87	0.58	49.1
	PH2	1.81	0.63	51.0
	RT1	1.36	0.16	7.9
	RT2	1.34	0.08	5.7
Spectrum Plant Total RNA Kit	ND1	1.90	0.94	154.5
	ND2	1.92	0.98	175.6
	PH1	1.81	0.86	62.6
	PH2	1.97	1.34	68.2
	RT1	1.79	0.82	27.5
	RT2	1.09	0.15	2.7
TRIzol	ND1	1.29	0.19	59.9
	ND2	1.13	0.16	83.4
	PH1	0.74	0.28	173.0
	PH2	0.92	0.20	146.4
	RT1	1.29	0.32	395.2
	RT2	1.26	0.24	253.8
TRIzol-column hybrid	ND1	1.34	0.35	9.8
	ND2	1.33	0.40	7.9
	PH1	1.01	0.21	10.9
	PH2	1.15	0.29	13.8
	RT1	1.27	0.43	13.7
	RT2	1.26	0.45	13.1
Modified CTAB	ND1	2.05	1.98	476.5
	ND2	2.06	2.16	578.7
	PH1	2.05	2.04	418.9
	PH2	2.05	1.99	685.1
	RT1	2.03	1.83	308.8
	RT2	2.05	1.88	347.8

**Table 2:** RNA sequencing data quality summary (reproduced from the NovoGene sequencing report).

Sample	Raw Reads (Gb)	Effective (%)	Error (%)	Q20 (%)	Q30 (%)	GC (%)
ND1	146,415,518	98.85	0.02	98.41	95.27	46.39
ND2	122,608,254	99.22	0.02	98.14	94.49	45.92
PH1	142,090,562	98.45	0.02	98.26	94.90	45.36
PH2	147,244,540	98.34	0.02	98.35	95.15	45.12
RT1	141,041,240	98.49	0.02	98.35	95.06	45.24
RT2	149,003,516	99.09	0.02	98.35	95.13	45.76

All the samples exhibit good sequencing results, with above average clean read ratios and clean read Q30 ratios. Raw reads: total amount of reads of raw data (Gb) for paired-end sequencing. Effective: (Clean reads/Raw reads) × 100%. Error: base error rate. Q20, Q30: (Base count of Phred value > 20 or 30)/(Total base count). GC: (G and C base count)/(Total base count).

## Data Availability

All data are provided in this manuscript. Sequencing data will be provided upon reasonable request to the corresponding author.

## Supplementary Materials

The following supporting information can be downloaded at: https://www.mdpi.com/article/10.3390/plants13030389/s1, Figure S1. The absorbance spectrum of RNA samples obtained from Norway spruce tissues through various extraction methods. The extraction methods include (A) RNeasy^®^ Plant Mini Kit (Qiagen), (B) Spectrum Plant Total RNA Kit (Sigma-Aldrich), (C) TRIzol method, (D) TRIzol-column hybrid method, and (E) modified CTAB method. The absorbance peaks observed at 220 nm (indicating organic contaminants), 260 nm (representing nucleic acids), and 280 nm (indicating proteins) serve as indicators of the quality and impurities present in RNA samples extracted using these five different methods.
