# Supplementary material for: An Optimized and Cost-Effective RNA Extraction Method for Secondary Metabolite-Enriched Tissues of Norway Spruce (Picea abies)
Source: Plants (Basel). 2024 Jan 28;13(3):389. doi: 10.3390/plants13030389 (PMC10857598; doi:10.3390/plants13030389)

Figure S1. The absorbance spectrum of RNA samples obtained from Norway spruce tissues through various extraction methods. The extraction methods include (A) RNeasy Plant Mini Kit (Qiagen), (B) Spectrum Plant Total RNA Kit (Sigma-Aldrich), (C) TRIzol method, (D) TRIzol-column hybrid method, and (E) modified CTAB method. The absorbance peaks observed at 220 nm (indicating organic contaminants), 260 nm (representing nucleic acids), and 280 nm (indicating protein) serve as indicators of the quality and impurities present in RNA samples extracted using these five different methods.

A. **RNeasy Plant Mini Kit**

Sample ID	Nucleic Acid	Unit	A260 (Abs)	A280 (Abs)	260/280	260/230	Sample Type	Factor
ND1	4,2	ng/µl	0,106	0,063	1,69	0,02	RNA	40,00

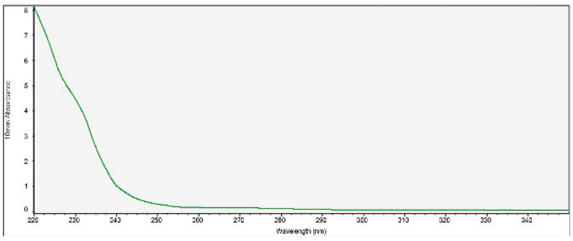

Sample ID	Nucleic Acid	Unit	A260 (Abs)	A280 (Abs)	260/280	260/230	Sample Type	Factor
ND2	5,3	ng/µl	0,133	0,079	1,68	0,09	RNA	40,00

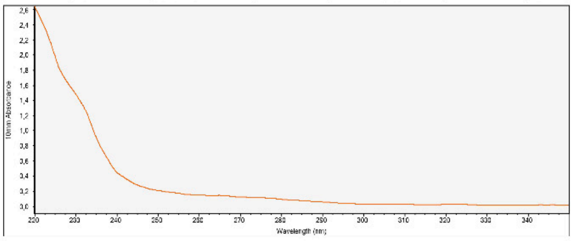

Sample ID	Nucleic Acid	Unit	A260 (Abs)	A280 (Abs)	260/280	260/230	Sample Type	Factor
PH1	49,1	ng/µl	1,229	0,657	1,87	0,58	RNA	40,00

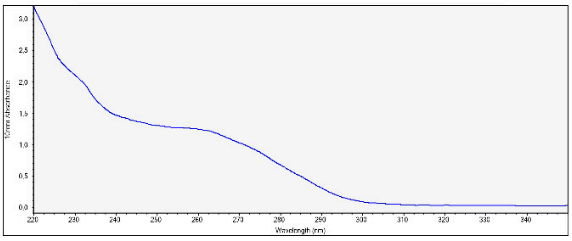

Sample ID	Nucleic Acid	Unit	A260 (Abs)	A280 (Abs)	260/280	260/230	Sample Type	Factor
PH2	51,0	ng/µl	1,276	0,705	1,81	0,63	RNA	40,00

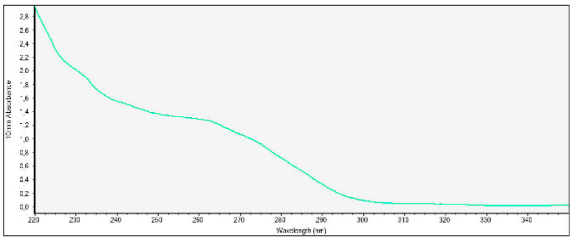

Sample ID	Nucleic Acid	Unit	A260 (Abs)	A280 (Abs)	260/280	260/230	Sample Type	Factor
RT1	7,9	ng/µl	0,197	0,145	1,36	0,16	RNA	40,00

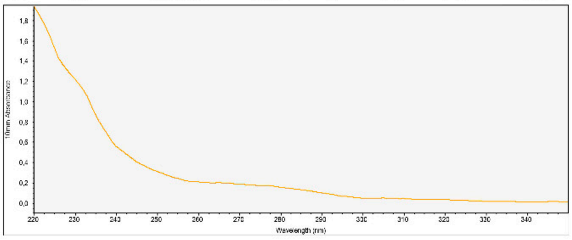

Sample ID	Nucleic Acid	Unit	A260 (Abs)	A280 (Abs)	260/280	260/230	Sample Type	Factor
RT2	5,7	ng/µl	0,143	0,107	1,34	0,08	RNA	40,00

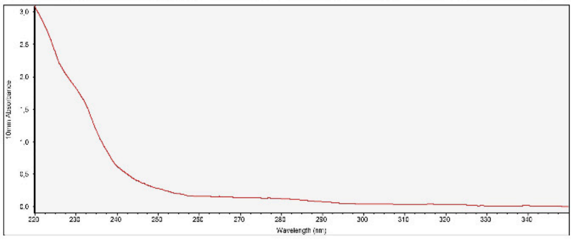

B.

Spectrum Plant Total RNA Kit

Sample ID	Nucleic Acid	Unit	A260 (Abs)	A280 (Abs)	260/280	260/230	Sample Type	Factor
ND1	154,5	ng/µl	3,862	2,031	1,90	0,94	RNA	40,00

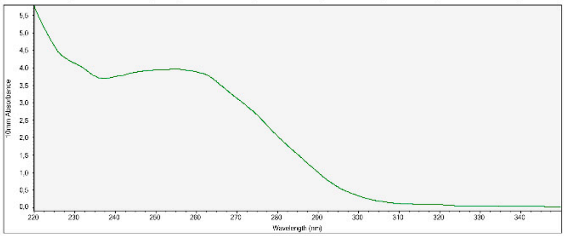

Sample ID	Nucleic Acid	Unit	A260 (Abs)	A280 (Abs)	260/280	260/230	Sample Type	Factor
ND2	175,6	ng/µl	4,390	2,290	1,92	0,98	RNA	40,00

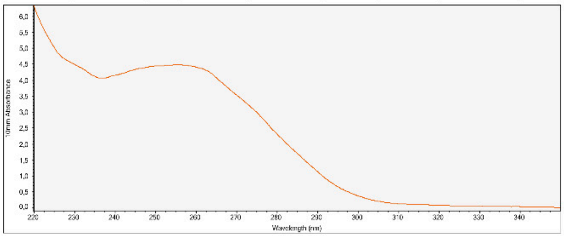

Sample ID	Nucleic Acid	Unit	A260 (Abs)	A280 (Abs)	260/280	260/230	Sample Type	Factor
PH1	62,6	ng/µl	1,566	0,864	1,81	0,86	RNA	40,00

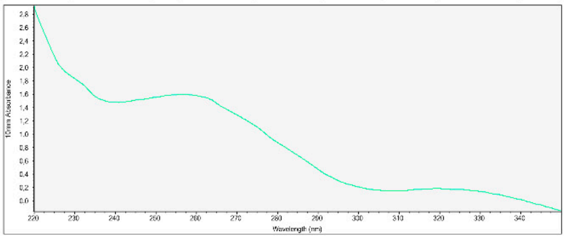

Sample ID	Nucleic Acid	Unit	A260 (Abs)	A280 (Abs)	260/280	260/230	Sample Type	Factor
PH2	68,2	ng/µl	1,704	0,863	1,97	1,34	RNA	40,00

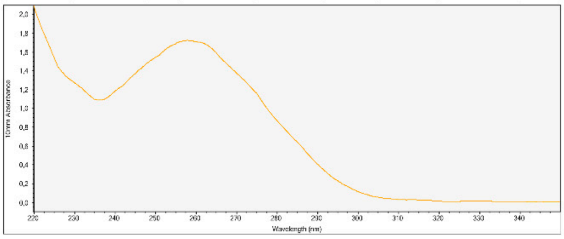

Sample ID	Nucleic Acid	Unit	A260 (Abs)	A280 (Abs)	260/280	260/230	Sample Type	Factor
RT1	27,5	ng/µl	0,689	0,385	1,79	0,82	RNA	40,00

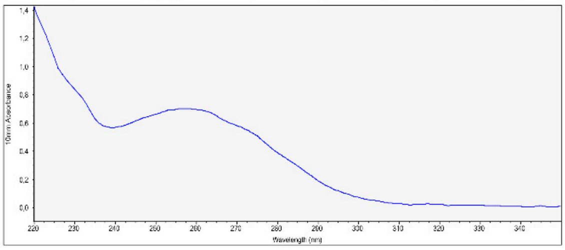

Sample ID	Nucleic Acid	Unit	A260 (Abs)	A280 (Abs)	260/280	260/230	Sample Type	Factor
RT2	2,7	ng/µl	0,068	0,063	1,09	0,15	RNA	40,00

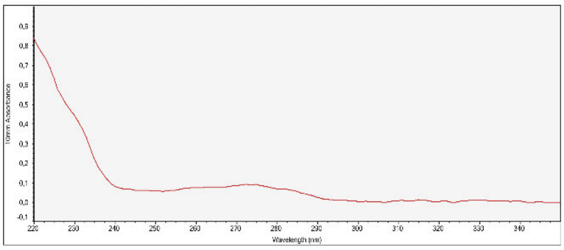

C. **TRIzol Method**

Sample ID	Nucleic Acid	Unit	A260 (Abs)	A280 (Abs)	260/280	260/230	Sample Type	Factor
ND1	59,9	ng/µl	1,498	1,160	1,29	0,19	RNA	40,00

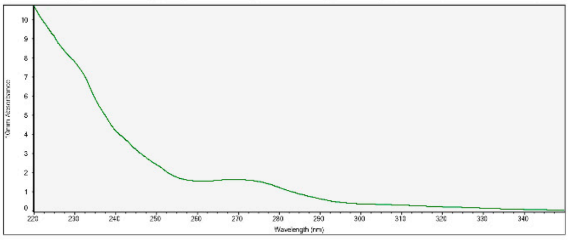

Sample ID	Nucleic Acid	Unit	A260 (Abs)	A280 (Abs)	260/280	260/230	Sample Type	Factor
ND2	83,4	ng/µl	2,086	1,849	1,13	0,16	RNA	40,00

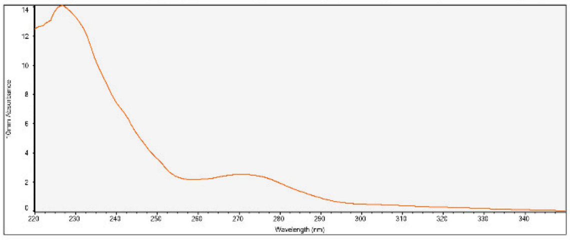

Sample ID	Nucleic Acid	Unit	A260 (Abs)	A280 (Abs)	260/280	260/230	Sample Type	Factor
PH1	173,0	ng/µl	4,325	5,811	0,74	0,28	RNA	40,00

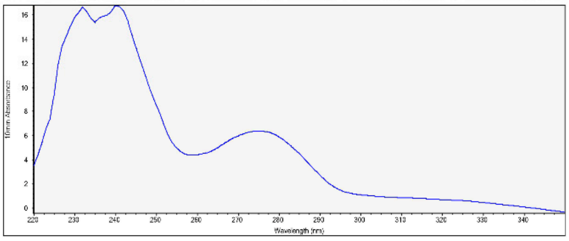

Sample ID	Nucleic Acid	Unit	A260 (Abs)	A280 (Abs)	260/280	260/230	Sample Type	Factor
PH2	146,4	ng/µl	3,661	3,972	0,92	0,20	RNA	40,00

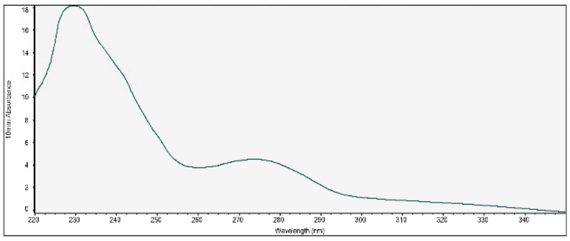

Sample ID	Nucleic Acid	Unit	A260 (Abs)	A280 (Abs)	260/280	260/230	Sample Type	Factor
RT1	395,2	ng/µl	9,881	7,617	1,30	0,28	RNA	40,00

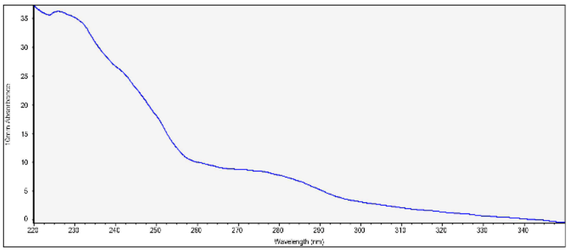

Sample ID	Nucleic Acid	Unit	A260 (Abs)	A280 (Abs)	260/280	260/230	Sample Type	Factor
RT2	253,8	ng/µl	6,345	5,018	1,26	0,24	RNA	40,00

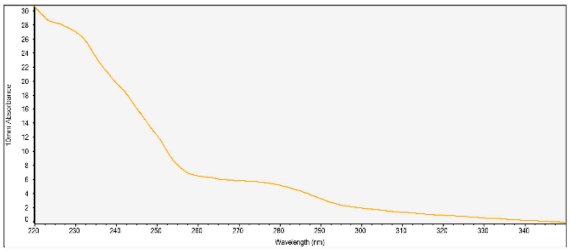

D.

TRIzol-column Hybrid Method

Sample ID	Nucleic Acid	Unit	A260 (Abs)	A280 (Abs)	260/280	260/230	Sample Type	Factor
ND1	9,8	ng/µl	0,245	0,182	1,34	0,35	RNA	40,00

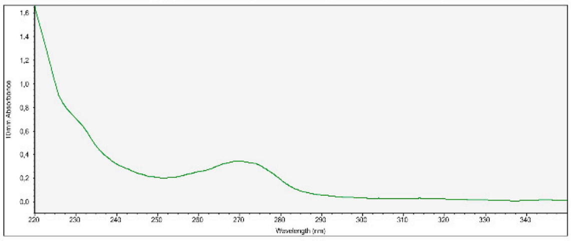

Sample ID	Nucleic Acid	Unit	A260 (Abs)	A280 (Abs)	260/280	260/230	Sample Type	Factor
ND2	7,9	ng/µl	0,197	0,148	1,33	0,40	RNA	40,00

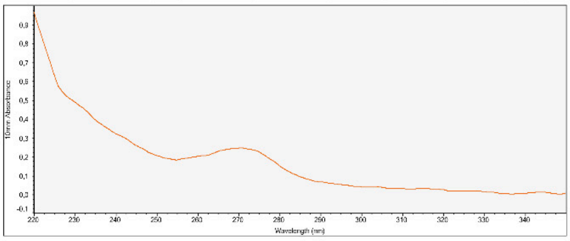

Sample ID	Nucleic Acid	Unit	A260 (Abs)	A280 (Abs)	260/280	260/230	Sample Type	Factor
PH1	10,9	ng/µl	0,273	0,270	1,01	0,21	RNA	40,00

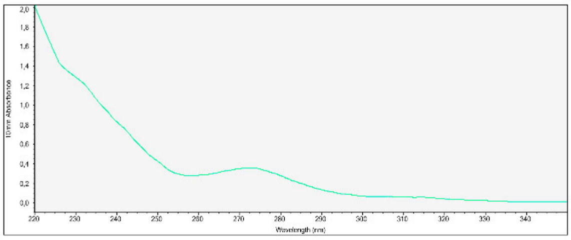

Sample ID	Nucleic Acid	Unit	A260 (Abs)	A280 (Abs)	260/280	260/230	Sample Type	Factor
PH2	13,8	ng/µl	0,345	0,300	1,15	0,29	RNA	40,00

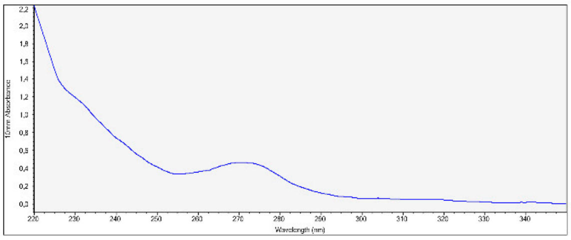

Sample ID	Nucleic Acid	Unit	A260 (Abs)	A280 (Abs)	260/280	260/230	Sample Type	Factor
RT1	13,7	ng/µl	0,341	0,268	1,27	0,43	RNA	40,00

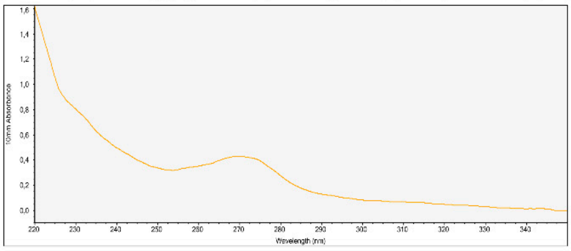

Sample ID	Nucleic Acid	Unit	A260 (Abs)	A280 (Abs)	260/280	260/230	Sample Type	Factor
RT2	13,1	ng/µl	0,328	0,260	1,26	0,45	RNA	40,00

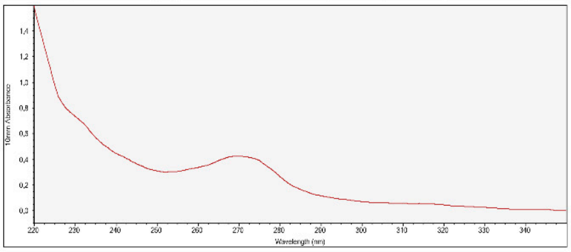

E.

CTAB Method

Sample ID	Nucleic Acid	Unit	A260 (Abs)	A280 (Abs)	260/280	260/230	Sample Type	Factor
ND1	636,3	ng/µl	12,727	6,209	2,05	1,96	RNA	40,00

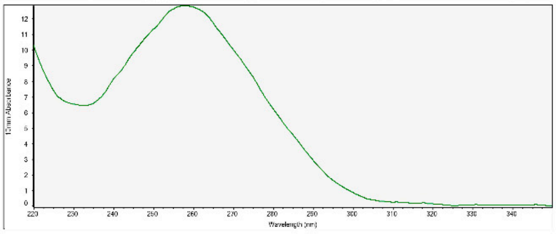

Sample ID	Nucleic Acid	Unit	A260 (Abs)	A280 (Abs)	260/280	260/230	Sample Type	Factor
ND2	682,8	ng/µl	13,656	6,559	2,08	2,22	RNA	40,00

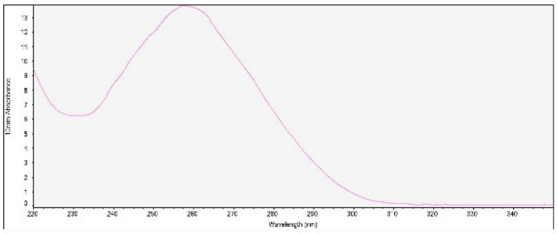

Sample ID	Nucleic Acid	Unit	A260 (Abs)	A280 (Abs)	260/280	260/230	Sample Type	Factor
PH1	366,9	ng/µl	7,339	3,565	2,06	2,01	RNA	40,00

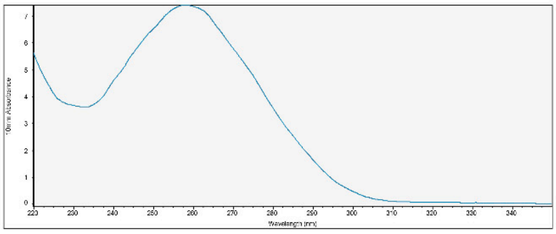

Sample ID	Nucleic Acid	Unit	A260 (Abs)	A280 (Abs)	260/280	260/230	Sample Type	Factor
PH2	471,2	ng/µl	11,779	5,433	2,17	4,35	RNA	40,00

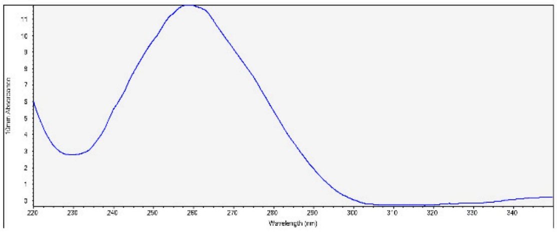

Sample ID	Nucleic Acid	Unit	A260 (Abs)	A280 (Abs)	260/280	260/230	Sample Type	Factor
RT1	370,1	ng/µl	9,253	4,522	2,05	1,90	RNA	40,00

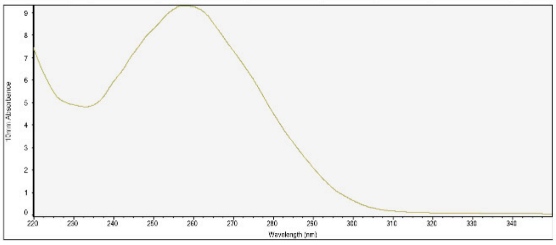

Sample ID	Nucleic Acid	Unit	A260 (Abs)	A280 (Abs)	260/280	260/230	Sample Type	Factor
RT2	261,7	ng/µl	6,541	3,196	2,05	1,88	RNA	40,00

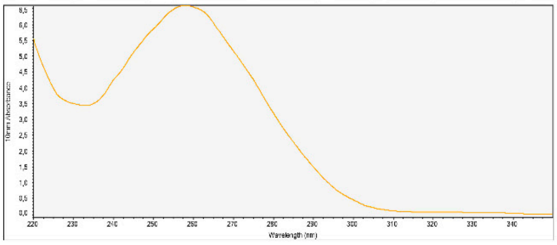

Supplement: Supplementary file 1 [file plants-13-00389-s001.zip › plants-2819469-supplementary.pdf]
